# Gamma-Glutamyl Transferase (GGT) Is the Leading External Quality Assurance Predictor of ISO15189 Compliance for Pathology Laboratories

**DOI:** 10.3390/diagnostics11040692

**Published:** 2021-04-13

**Authors:** Brett A. Lidbury, Gus Koerbin, Alice M. Richardson, Tony Badrick

**Affiliations:** 1 The National Centre for Epidemiology and Population Health, Research School of Population Health, The Australian National University, Canberra, ACT 2601, Australia; brett.lidbury@anu.edu.au (B.A.L.); alice.richardson@anu.edu.au (A.M.R.); 2 Faculty of Health, University of Canberra, Canberra, ACT 2617, Australia; guskoerbin@gmail.com; 3 Statistical Consulting Unit, Australian National University, Canberra, ACT 2601, Australia; 4 Australasia Quality Assurance Programs, Royal College of Pathologists, St. Leonards Sydney, NSW 2065, Australia

**Keywords:** ISO 15189, external quality assurance, pathology, machine learning and prediction

## Abstract

Pathology results are central to modern medical practice, informing diagnosis and patient management. To ensure high standards from pathology laboratories, regulators require compliance with international and local standards. In Australia, the monitoring and regulation of medical laboratories are achieved by conformance to ISO15189-National Pathology Accreditation Advisory Council standards, as assessed by the National Association of Testing Authorities (NATA), and an external quality assurance (EQA) assessment via the Royal College of Pathologists of Australasia Quality Assurance Program (RCPAQAP). While effective individually, integration of data collected by NATA and EQA testing promises advantages for the early detection of technical or management problems in the laboratory, and enhanced ongoing quality assessment. Random forest (RF) machine learning (ML) previously identified gamma-glutamyl transferase (GGT) as a leading predictor of NATA compliance condition reporting. In addition to further RF investigations, this study also deployed single decision trees and support vector machines (SVM) models that included creatinine, electrolytes and liver function test (LFT) EQA results. Across all analyses, GGT was consistently the top-ranked predictor variable, validating previous observations from Australian laboratories. SVM revealed broad patterns of predictive EQA marker interactions with NATA outcomes, and the distribution of GGT relative deviation suggested patterns by which to identify other strong EQA predictors of NATA outcomes. An integrated model of pathology quality assessment was successfully developed, via the prediction of NATA outcomes by EQA results. GGT consistently ranked as the best predictor variable, identified by combining recursive partitioning and SVM ML strategies.

## 1. Introduction

The introduction of the ISO 15189 International Standard is regarded as an advance for the maintenance of quality in laboratory medicine, providing a “Gold Standard” that applies to technical and management performance, while emphasizing the value of the duties undertaken by laboratory professionals [1,2]. Alongside these standards are additional discussions on quality improvement [3,4], suggesting a dynamic, ongoing process of clinical laboratory quality assurance.

In Australia, National Pathology Accreditation Advisory Council (NPAAC) Standards are the requirement for medical laboratories, with periodic assessment of compliance the responsibility of NATA [5]. Complementing this approach, an external quality assurance (EQA) scheme is run by the Royal College of Pathologists of Australasia-Quality Assurance Programs (RCPAQAP). These two distinct processes are connected in the pursuit of the assessment of quality pathology performance, but traditionally have not been integrated into a quantitative model of quality monitoring. Drawing upon integrated NATA and EQA results will create opportunities for advances in the assessment of laboratory quality via predictive modelling, and performance monitoring improvements.

The NPAAC standards and Guidelines are Australian legislated requirements for medical laboratories to be accredited to obtain reimbursement for testing. The standards encompass ISO 15189 and include requirements for governance, supervision, ethical practice, facilities, staffing and communication, which are assessed by NATA. The suite of standards also includes technical requirements for retention of samples, molecular testing, cytological screening, IVD validation and many other technical requirements. The RCPAQAP EQA provider is accredited to ISO 17043, and uses ISO 13528 as an adjunct standard for the statistical evaluations of EQA results.

We previously reported preliminary results on the integration of NATA and EQA results, which included the results of a systematic scoping review of the clinical laboratory quality literature [6]. Data modelling via machine learning algorithms was conducted on NATA and EQA data provided by a state-wide, public pathology network. The aim of the model was the prediction of NATA performance by EQA results, determined from the number of NATA compliance conditions reports, linked to EQA marker relative deviation, as determined by the extent of variation from specific target values [6]. It must be noted that the investigation of EQA markers (e.g., ALT) in the context of this study did not concern their physiological or diagnostic roles, but only their function as EQA accuracy indicators.

This study further explores the relationship between NATA and EQA results [6] through a larger laboratory sample from a separate quality assurance cycle, and which applied additional machine learning methods. Furthermore, the results confirmed gamma-glutamyl transferase (GGT) as the leading predictor of NATA assessment outcome, and proposes a method to detect similarly strong EQA predictive markers.

## 2. Methods

### 2.1. Data

Data provided for this research project were obtained directly from a pathology network, covering hundreds of laboratories and collection centers within a specific Australian state jurisdiction. Because of the confidentiality requirements of the two processes, neither the RCPAQAP nor NATA were involved in the acquisition of data.

The study sample comprised ten General (G) and 29 Branch (B) designated laboratories, for a total sample of 39 pathology laboratories. The G designation is for larger multi-discipline laboratories, with the B designation for branches of G laboratories with G supervision, as per NPAAC definitions [7]. The branch laboratories are related to the general laboratory in a hub (supervisory) and spoke (regional) model.

The EQA results obtained from the RCPAQAP covered an assessment period comprising 16 individual testing challenges (mean of two samples) over a four-month period during 2017. A range of routine and second tier EQA markers were provided, with the eventual modelling utilizing serum creatinine plus electrolytes (Na^+^, K^+^, Cl^−^, bicarb, Ca^++^, PO_4_, Mg^++^), or liver function test (LFT) markers: Alanine aminotransferase (ALT), Aspartate aminotransferase (AST), gamma-glutamyl transferase (GGT), Lactate dehydrogenase (LD), Alkaline phosphatase (ALP), total protein (TP), Albumin (Alb) and total bilirubin (TBil). These markers featured for each laboratory, and had the largest sample sizes, hence their choice for modelling against NATA response classes. Full Blood Count (FBC) data were not available.

NATA inspections were also conducted during 2017, with one compliance report provided for each laboratory, which presented written feedback on performance, as well as “conditions” stipulated to meet the required standards under ISO 15189 (see the following section).

#### 2.1.1. NATA Results and Response Classes

The results of NATA site audits utilized in this study included three types of advice in relation to performance measured against ISO 15189 compliance—namely Conditions (C), Minor (conditions) (M) and Observations (O). A NATA Condition is an assessment that requires immediate attention and resolution before the following inspection. If not addressed adequately, Conditions can lead to laboratory closure or other NATA sanction. Minor (conditions) require attention, which if not addressed, with risk becoming a full Condition during the following evaluation. Observations (O) are general feedback on laboratory operations that are not linked to condition requirements.

As recommended previously [6], response (NATA) classes were established from the number of Minor (M) condition reports per laboratory to represent the extent of ISO 15189 compliance. To develop the NATA response classes, Minor medians were calculated from the pooled laboratory data (*n* = 39), and each laboratory was classified as high (H) or low (L) depending upon whether their Minor total fell above or below the medians calculated from all laboratories in the sample; therefore, 50% of laboratories in the sample were allocated to class H, or class L. Laboratories with the exact median value were allotted to the high or low response classes depending on whether the laboratory concerned also attracted any Condition (C) reports (allocated to high), while laboratories that attracted no C reports received a low response classification.

The placement of a laboratory in the high NATA class represented poorer ISO 15189 compliance (as reflected by more than the median number of reports), whereas the low class represented the better performing laboratories in relation to compliance. Some low NATA class laboratories recorded zero Condition or Minor reports, representing the best performance in terms of ISO 15189 compliance.

#### 2.1.2. Calculation of Relative Deviation for EQA Markers

**Figure 1:**
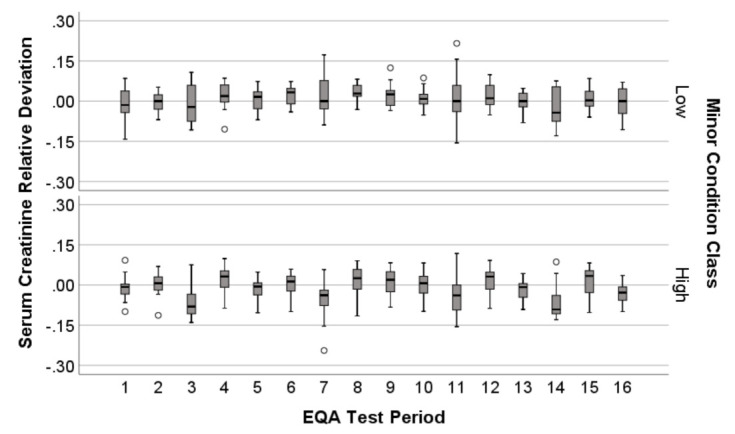
External Quality Assurance (EQA) relative deviation presented as boxplots for serum creatinine, separated by National Association of Testing Authorities (NATA) Minor class Condition reports for comparison, over the entire Royal College of Pathologists Australasia Quality Assurance Program (RCPAQAP) quality assurance cycle comprising 16 separate test challenges (4-months).

The results for each marker across all 16 EQA challenges of the cycle were transformed to relative deviation scores, thus representing how close the laboratories were to achieving the EQA target value for the specific marker at each specific testing point (1–16) (Figure 1). The target value was determined from the EQA mean reference value calculated from the national database comprising 450–600 laboratories. The equation to calculate relative deviation from EQA results is—

$$\mathrm{EQA}\;\mathrm{Relative}\;\mathrm{Deviation}=\frac{\left(\mathrm{Individual}\;\mathrm{Lab}\;\mathrm{result}-\mathrm{EQA}\;\mathrm{Target}\;\mathrm{value}\right)}{\mathrm{EQA}\;\mathrm{Target}\;\mathrm{value}}$$

A relative deviation result of zero (0.0) was a perfect EQA outcome; namely, the exact EQA target value was achieved. Relative deviation results that did not achieve exact agreement were represented as negative (−) or positive (+), indicating a laboratory EQA result below or above the EQA target value, respectively, with the degree of variation from zero on a continuous scale, indicating the extent of variation from the target value.

All EQA data were applied to machine learning models, and statistical analyses and data plots were first transformed into relative deviation values, as achieved via the above equation. Therefore, all results and conclusions reported here reflect variation in achieving EQA target values for the predictive markers interrogated, and analyzed against NATA response class.

EQA results were collected from each of the 39 laboratories and for all 2017 challenges, with the 2017 NATA assessor reports linked thereafter to each participating laboratory. Because the EQA process provided multiple results, whereas NATA provided a single result, the same NATA response class allocation (Minor-high or low) was attached to each of the 16 EQA results prior to analysis (Figure 1).

#### 2.1.3. Direct Comparison of EQA and NATA Results

Laboratories were ranked in order from best to worst EQA performance for each marker as calculated by coefficient of variation percent (CV%). The CV% was calculated for individual laboratories from the 16 challenges across the 2017 EQA cycle. This within-laboratory CV% thereafter provides performance rankings, which were clustered into quartiles (Table 1). Quartile 1 represented the best EQA performance, and quartile 4 the worst performance, from the laboratories investigated, and for each individual EQA marker assessed.

The CV% was determined by the routine calculation as the SD divided by the average/mean of the participant’s range of concentrations, expressed as a percentage mid-point average (full details—Supplementary Materials).

Mean numbers ($\bar{x}$ ± SEM) of NATA C and M conditions were calculated for each quartile, and compared directly to quartile rankings to determine whether NATA and EQA performance relationships were obvious. To demonstrate this direct comparison, GGT, serum creatinine and potassium were examined (Table 1).

#### 2.1.4. Distribution Profile of EQA Performance

To visualize the relative deviation of EQA markers over the 16 challenges, results were summarized by boxplots displaying median and inter-quartile range (IQR) results, in the context of NATA Minor class (Figure 1).

Included in the distribution analyses were histograms for GGT and TP, which included the Kolmogorov–Smirnov (K–S) two-sample test. The K–S test was applied to test the hypothesis that there were no significant differences in frequency distribution for GGT and TP when separated into NATA High and Low Classes.

### 2.2. Statistical Methods

Standard statistical analyses that included the calculation of sample mean, median, standard deviation, histograms and other plots, and two-sample Kolmogorov–Smirnov tests (K–S), were performed using SPSS for Windows (version 26) [8].

For the assessment of machine learning model prediction efficacy, Kappa [9] and McNemar’s [10] statistics were used. Both statistics estimate the degree of agreement in data arranged into 2 × 2 contingency tables, relevant here for the statistical comparison of observed and predicted NATA Classes in the test data set.

A *p*-value of 0.05 was applied to decide statistical significance, unless stated otherwise.

### 2.3. Machine Learning-Integration of NATA and EQA Results

The R Statistical programming language (version 3.6.1) was used for all machine learning analyses, and ultimate construction of NATA-EQA prediction models [11]. The R package e1071 was used for support vector machine (SVM) modelling [12,13]. The e1071 package supported pre-model tuning for cost and gamma coefficients to ensure model optimization. For recursive partitioning, two algorithms were employed; (i) the rpart package for single decision trees [14], and (ii) the randomForest (RF) package for analyses of the same name [15].

The R caret package was used for the tuning and inspection of machine learning models, particularly for random forests [16]. This package supported the tuning of RF models via the mtry parameter, according to receiver operating characteristics (ROC). As well as model fitting, caret assessed model accuracy and efficacy. For RF, the aforementioned Kappa and McNemar’s statistics were calculated to allow the assessment of model robustness.

Training-testing ratios were set at a 70–30% data split for analyses via caret, with the final results calculated from the 30% testing sub-sample. RF used 5000 trees (*ntree* = 5000) for each analysis, with the mtry value (number of trees per decision node) determined for each model prior to training. The replace function was also included to provide randomization of data entry into the models [15,16].

As well as supervised RF models to predict NATA Class via EQA results, an unsupervised model was tuned and conducted that evaluated the variation of all EQA markers relative to each other, without reference to the NATA response, and as an indication of predictive potential.

Rpart (decision tree) analyses were conducted on a minimum node split of 30 observations, with complexity parameters (cp) applied according to pre-model tuning.

The models developed involved the prediction of NATA class (high or low categories) by multiple EQA relative deviation scores. Therefore, the NATA class acted in the model as the response to be predicted by EQA relative deviation. The SVM models included only the top 2–3 EQA predictors identified from RF model tuning and optimization, while the final RF model of EQA-ISO15189 integration included ALT, AST, GGT, sodium bicarbonate and serum creatinine relative deviations as predictor variables.

## 3. Results

The following results were determined by machine learning models to predict NATA outcomes via a range of EQA markers. The NATA outcomes reflected ISO15189 compliance by laboratories over a two-year period, represented as two separate classes (categories). All EQA results were calculated as a relative deviation value prior to inclusion in the models as predictor variables, as well as supporting plots and statistics.

### 3.1. Direct Relationship of EQA Performance to NATA (ISO15189) Compliance

During the 2017 assessment period for the 39 laboratories investigated, 128 Conditions (C) and 244 Minor (M) conditions were recorded in total from NATA assessments. How the number of NATA conditions reflected EQA performance over the same assessment period was central to the following investigations.

Quartile rank for each laboratory (as decided by within-laboratory CV%) was determined for GGT, serum creatinine and serum potassium, and matched with the mean ($\bar{x}$) number of NATA C, C+M and M reports (Table 1). NATA performance was not reflected by the EQA quartile rankings for GGT, creatinine nor potassium. Therefore, the direct matching of EQA performance rankings and NATA results did not show an obvious relationship, hence the more sophisticated investigation of the combined 2017 NATA and EQA results.

### 3.2. Arrangement of EQA Results in Relation to NATA Response

Boxplots representing the entire EQA cycle (16 test challenges over four months of 2017) were plotted for serum creatinine, as an example EQA marker included in the following machine learning studies (Figure 1).

As presented by Figure 1, serum creatinine relative deviation fluctuated over the 16 EQA challenges, both between EQA challenges and when separated by NATA class. This is the graphical representation of how distinct NATA and EQA results were arranged for the machine learning investigations that follow, with NATA results matched to an EQA cycle that comprised 16 individual challenges per marker. NATA assessments were conducted once every two years, and reported on system-level performance across a number of management and technical ISO15189 clauses, representing long-term performance of the complex systems that underpin day-to-day laboratory performance and, which if Conditions were detected, required lengthy periods to ameliorate.

### 3.3. Machine Learning-Predicting NATA Compliance by EQA Relative Deviation

#### 3.3.1. Creatinine and Electrolytes

NATA-EQA recursive partitioning and SVM modelling explored EQA relative deviation in creatinine and electrolytes (with and without GGT) to predict NATA response class.

**Figure 2:**
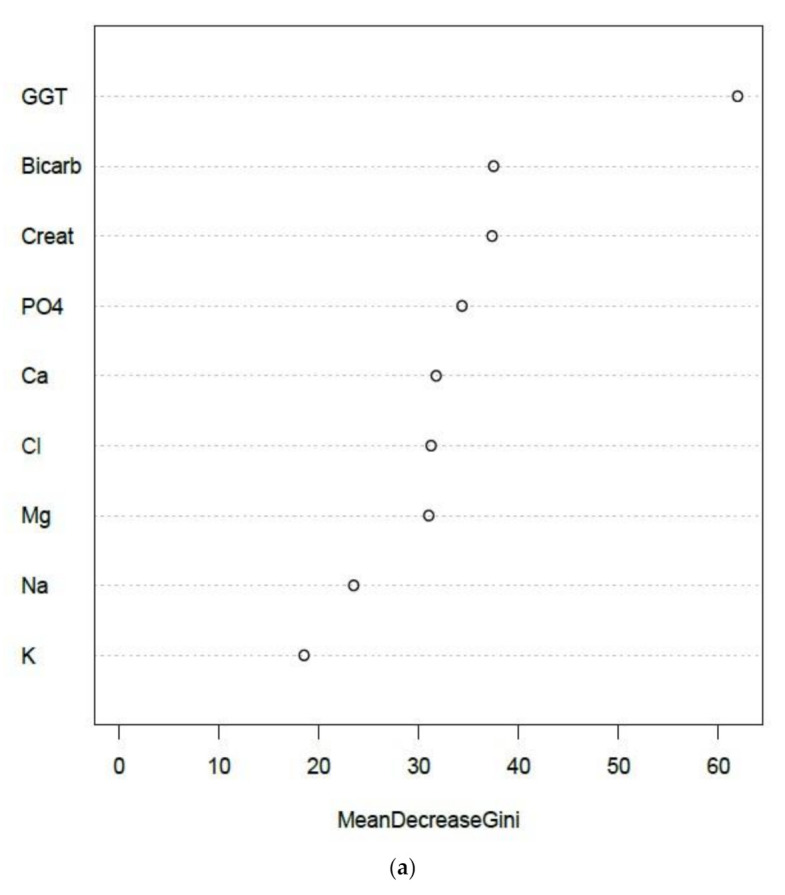

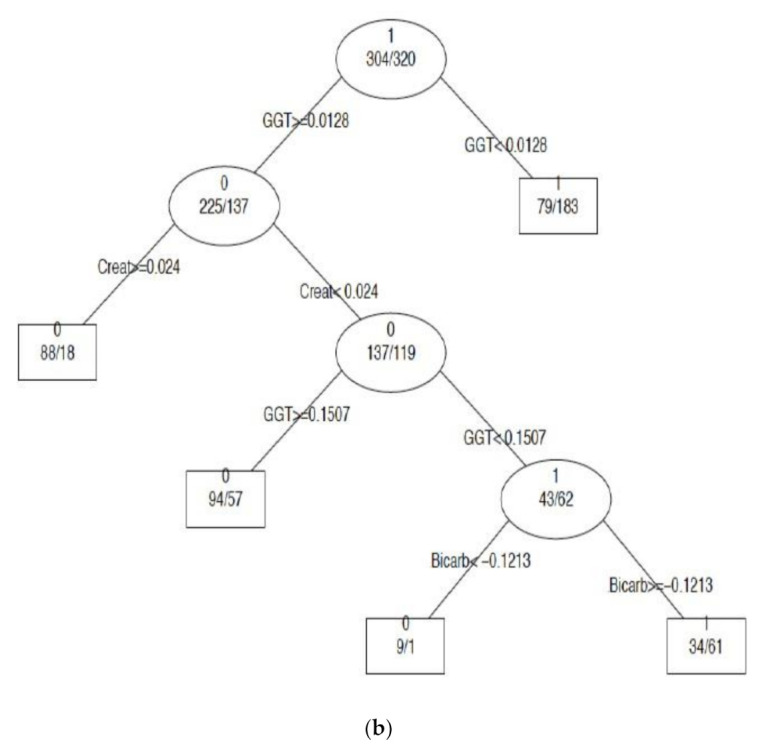

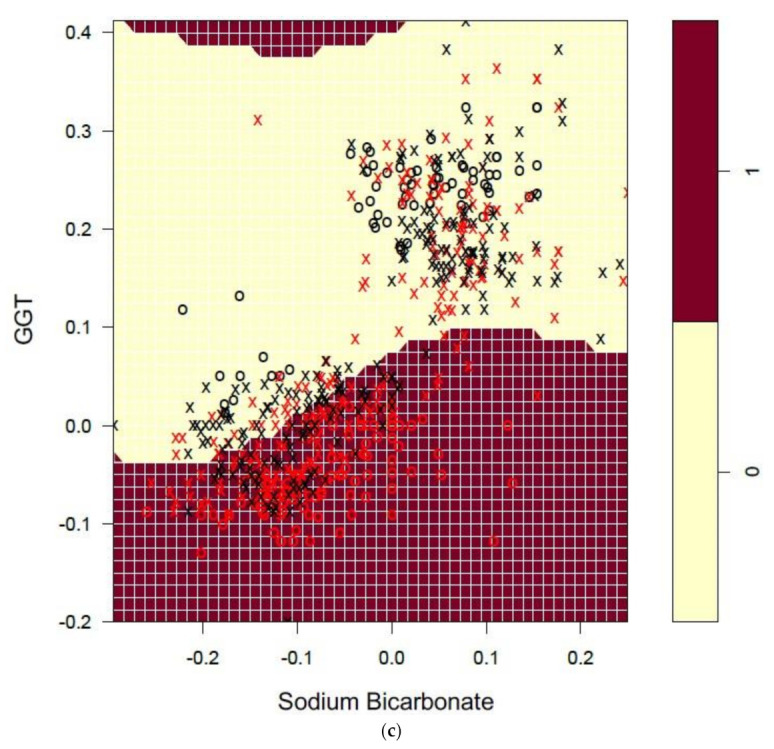
Plots summarizing the recursive partitioning and support vector machine (SVM) analyses of EQA relative deviation prediction of NATA Minor Class Condition reports by electrolyte-creatinine data (and GGT). (**a**) Random Forest; (**b**) Single Decision Tree and (**c**) SVM model. Features of the random forest modelling are presented in Table 2. For SVM, only GGT and sodium bicarbonate were included in the final model. Low NATA Class (Yellow); High NATA Class (Red). Model accuracy (10-fold cross validation)-66.23% (58.1–72.6%), at coefficients of Cost = 4, Gamma = 0.125 (C-classification and radial kernel). X denotes the support vectors (that define the calculated hyperplane required to perform as a classification model). O symbols denote the observations (Red is a class 1 prediction, and Black a class 0 prediction). GGT = gamma-glutamyl transferase.

Integrated NATA-EQA recursive partitioning models are summarized in Figure 2 and supported by Table 2. GGT, serum bicarbonate and creatinine were the leading predictors of NATA class. Bicarbonate and creatinine retained their leading predictor ranking when GGT was removed from the random forest (RF) model (result not shown). The importance of GGT, creatinine and bicarbonate relative deviations as leading predictors of NATA class were confirmed by decision tree (Figure 2b). The removal of GGT weakened RF model accuracy, particularly the prediction of the low (M) NATA class, and models for both NATA classes were less accurate without GGT (Table 3).

Decision tree prediction accuracy (%) broadly agreed with the random forest results (Table 2), but were higher at some nodes, e.g., GGT ≥ 0.0128 + creatinine ≥ 0.024 ⇒ 83.02%. Random forest and decision tree results are best considered in tandem when determining predictive rules.

The RF model had an overall accuracy prediction of 71%, with both positive and negative predictive values over 70% (Table 2). Sensitivity was approximately 9–14% points higher than specificity, indicating greater success at correctly predicting true positive results across high and low classes. The Kappa statistic was 42%, suggesting moderate performance in agreement between observed and predicted outcomes. The McNemar’s test was non-significant (*p* = 0.272), indicating no evidence of a difference in classification.

The top two RF NATA class predictors were explored by support vector machine (SVM), illustrating the interaction patterns between the leading EQA predictors of NATA class (Figure 2c). The kinetics of GGT in relation to bicarbonate demonstrated the sensitivity of GGT for NATA class prediction. In predicting the NATA high class (1), bicarbonate remained consistent across the range, while GGT fluctuated from < 0.0 to 0.1 relative deviation with increasing bicarbonate values, representing approximately a 10–12% variation in GGT response to fluctuations in bicarbonate accuracy. This SVM model, with only two EQA predictor variables, had an accuracy of 66.23% (Cost = 4; Gamma = 0.125).

#### 3.3.2. Liver Function Tests (LFTs)

GGT was confirmed as a powerful predictor within a serum electrolyte profile [6], so GGT performance with other LFTs was of interest. The EQA data available covered the LFT range of routine chemical pathology markers.

**Figure 3:**
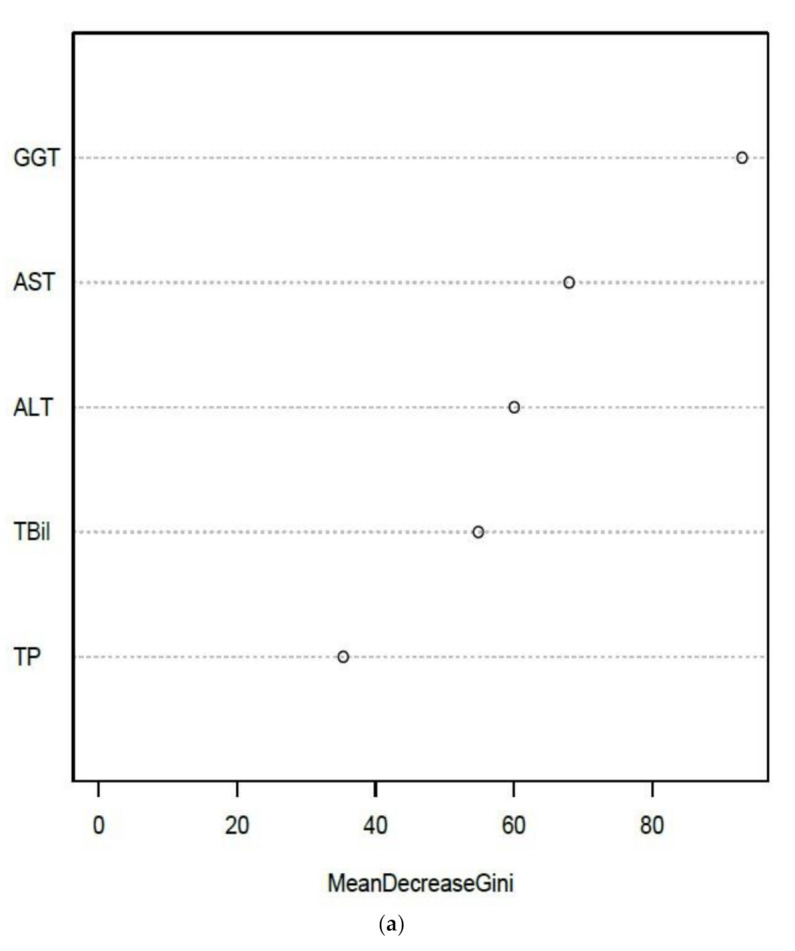

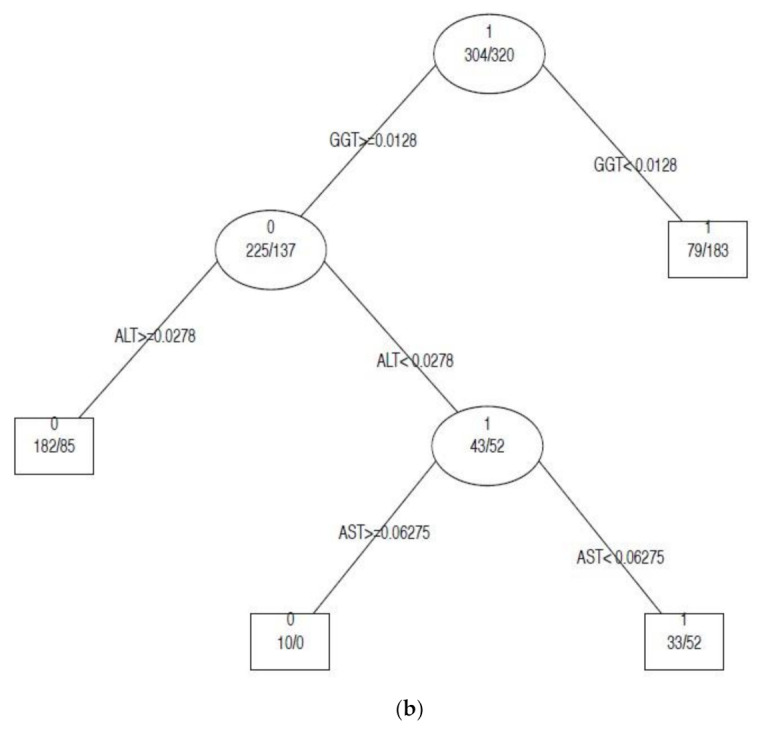
Recursive partitioning results from the modelling of EQA Liver Function Test (LFT) relative deviation results to predict the NATA Minor Class Condition reports. (**a**) Random Forest model predicting NATA Classes; (**b**) the same model as (**a**), but interrogated by a single decision tree. Features of the random forest modelling are presented in Table 4.

Amongst the EQA results for the selected LFTs, GGT was the leading predictor in the RF models, which was confirmed by a single decision tree analysis (Figure 3a,b). AST and ALT deviations were the second and third ranked RF predictors, respectively, but were inverted for the single decision tree. The RF model had positive and negative predictive values over 70%, a Kappa statistic result of 43%, a McNemar’s *p*-value of 0.27 and an overall model accuracy of 72% (Table 4), which suggested a robust prediction model.

**Figure 4:**
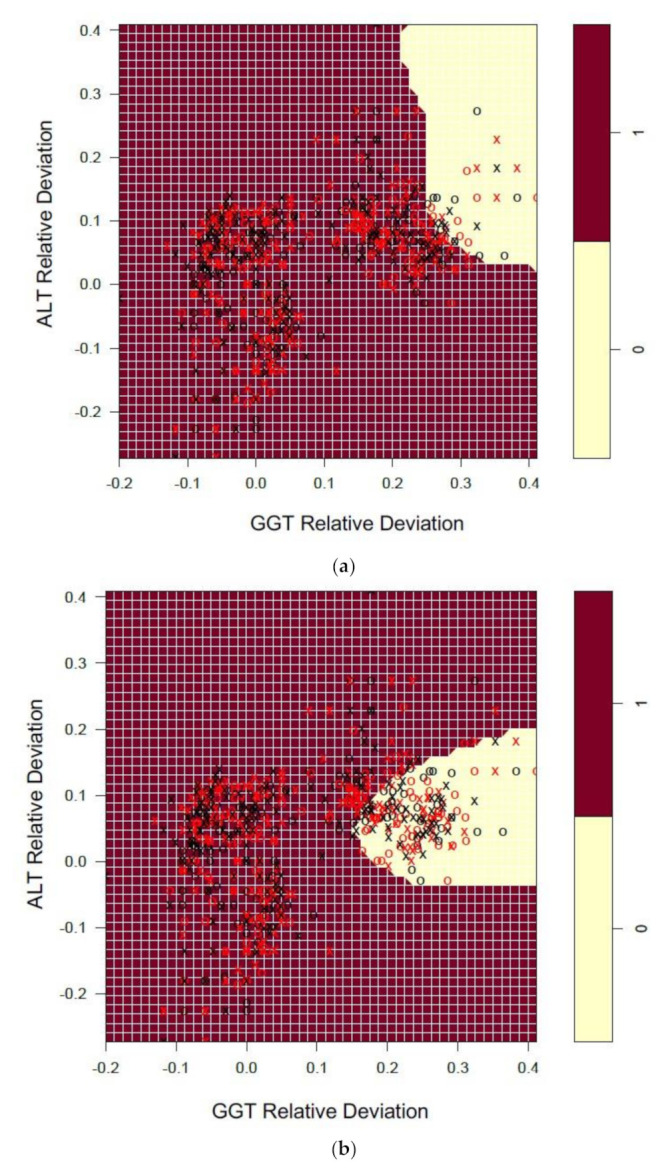

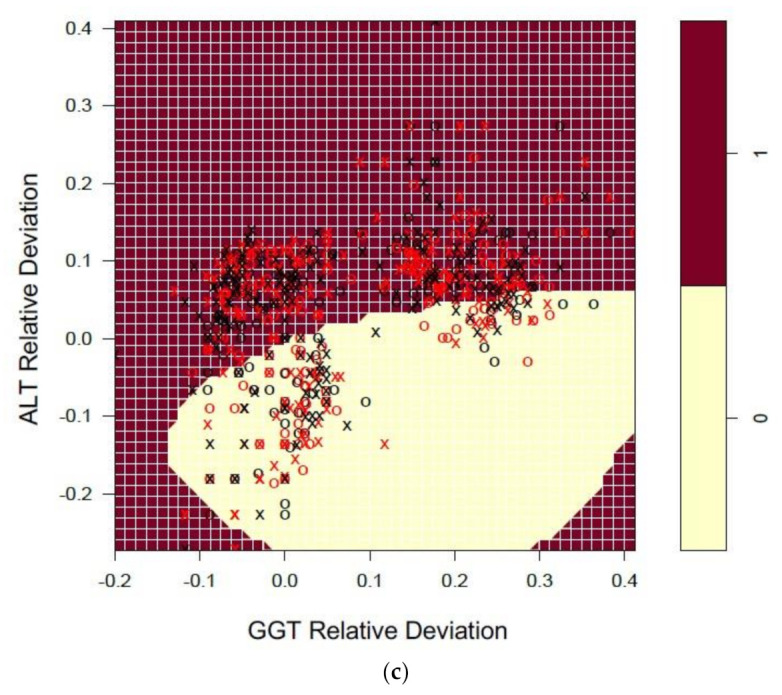
Support vector machine (SVM) plots representing the interactions between the top three EQA Liver Function Test (LFT) predictors of NATA Minor Class Condition reports (High = Red; Low = Yellow). The plots represent ALT and GGT relative deviations on the *x-y* axes, with SVM slices for AST included at the following relative deviation values: (**a**) −0.15 (**b**) 0.0 and (**c**) 0.15. The SVM model ran the liver function test relative deviations ALT, AST, Total Bilirubin, GGT, LD and Total Protein (C-classification and radial kernel; Cost = 2, Gamma = 0.25). Model accuracy (10-fold cross validation) –73.01% (65.96–79.17%). X denotes the support vectors (that define the calculated hyperplane required to perform as a classification model). O symbols denote the observations (Red is a class 1 prediction, and Black a class 0 prediction).

Informed by the recursive partitioning results, SVMs focused on the prediction of NATA Minor Classes via the top three EQA predictor variables, GGT, ALT and AST, from the full model that included all LFT markers (Figure 4).

With GGT and ALT *x* and *y* axis variables, an extra dimension was added to the SVM modelling via AST slices spanning negative to positive relative deviations (Figure 4a–c), unlike the two variable SVM model described earlier. The impact of changing the AST relative deviation from −0.15 to 0.15 had a clear influence on the prediction of the NATA class (Category 0-Low, yellow region), as observed by GGT and ALT. At −0.15 and 0.0 AST, both GGT and ALT reflected predominantly positive EQA relative deviations, while at AST 0.15 the range of the NATA low class extended towards negative GGT and ALT dimensions, with GGT restricted to approximately 0.0–0.3 at an ALT relative deviation of-0.4, while expanding its range towards 0.0 ALT (Figure 4c).

NATA class prediction accuracy for SVM was similar to that found for the recursive partitioning models, at 73.01% (10-fold cross validation; range 65.96–79.17%). Repeating the SVM with only the top three EQA predictors (GGT, AST, ALT) resulted in a drop of 10-fold cross validation accuracy to 67.48% (62.90–77.42%), but still within the range of recursive partitioning prediction accuracy.

#### 3.3.3. NATA Class Prediction by Combined Creatinine-Electrolyte-LFT EQA Results

To explore whether the prediction of NATA classes could be improved, the leading predictors from the separate creatinine-electrolyte and LFT models were combined into a single random forest model (final model).

**Figure 5:**
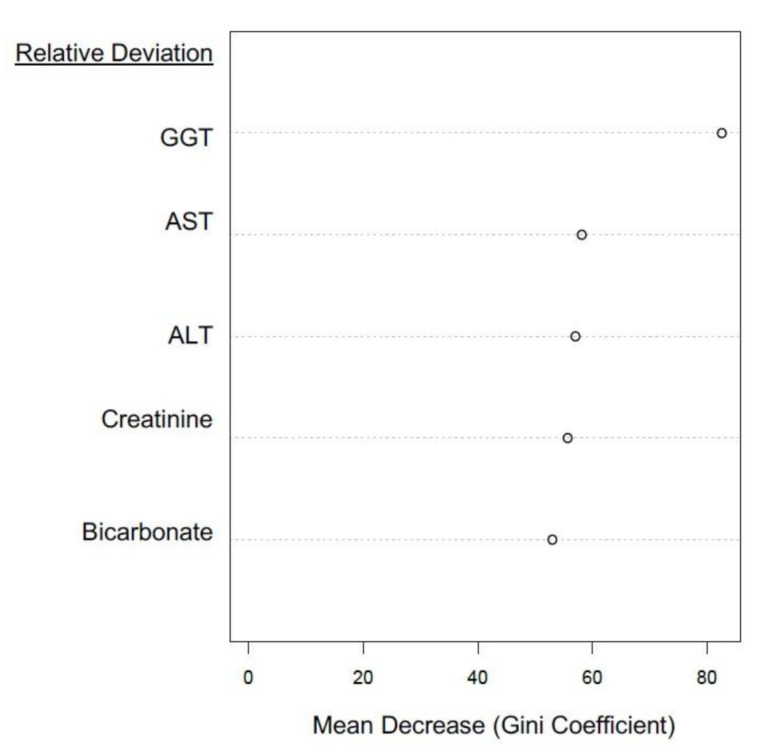
Random Forest (recursive partitioning) analyses that interrogated a combined serum creatinine-electrolyte and LFT (EQA relative deviation) model to determine the best predictors of NATA class (NATA Minor-High or Low Condition reports). The analysis used leading predictors from separate models (Figure 2 and Figure 3), namely, GGT, AST, ALT, creatinine and sodium bicarbonate. Tuning and the predictive features of the random forest modelling are presented in Table 5.

Figure 5 confirmed the position of GGT as the leading predictor variable, AST and ALT ranked second and third respectively, with creatinine and bicarbonate the least powerful predictors of NATA Classes (but still impressive at between 50–60 on the Gini Index). Full blood count (FBC) EQA results were not available for inclusion, although investigation of NATA-EQA results from another laboratory network identified red cell distribution width (RDW) as leading predictor of NATA M class from the FBC profile (results not shown).

The combined EQA marker model accuracy and prediction values (Table 5) were not superior to the separate creatinine-electrolyte and LFT models (Table 2 and Table 4).

The RF final model of NATA response was repeated by logistic regression (LR), as a comparison prediction method. The pseudo-R^2^ result (Nagelkerke) was less than 20%, with a Hosmer-Lemeshow result of *p* = 0.05, suggesting a poor model fit. Further investigation of the LR model identified issues of collinearity, particularly for bicarbonate and ALT deviations, rendering the results unreliable.

To conclude, LFT or creatinine-electrolyte EQA predictors will suffice for the prediction of NATA compliance performance, providing that GGT is included. As noted previously, removing GGT from the creatinine-electrolyte model resulted in a 5.1% predictive accuracy decrease (Table 3).

#### 3.3.4. Investigations of EQA Marker Predictive Capacity via Unsupervised Analyses

To further investigate the variation of the EQA markers applied to predict NATA performance, unsupervised analyses of EQA markers were conducted without reference to NATA class.

**Figure 6:**
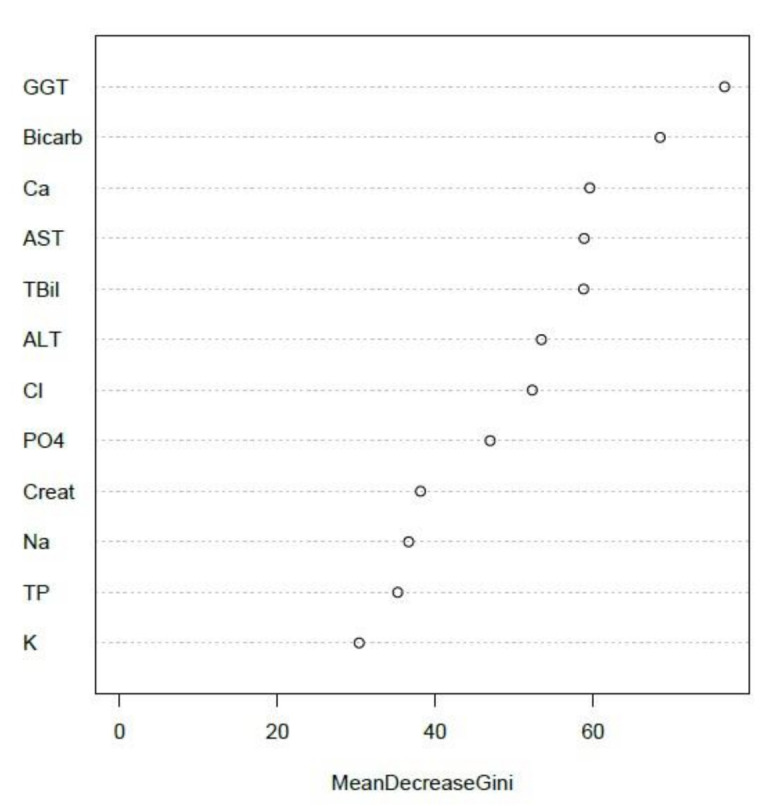
Ranking of EQA markers as calculated by an unsupervised random forest model (no NATA Class response included). The model includes the relative deviation values of all markers assessed over 16 EQA challenge cycles conducted over 4 months (2017) from 39 laboratories (*n* = 624).

Unsupervised random forest ranked GGT as the top EQA marker, with bicarbonate second, and AST in the top five (Figure 6), reflecting the final supervised model summarized by Figure 3. Additionally, potassium (K) and total protein (TP) were the two lowest ranking EQA markers, consistent with supervised models of electrolytes and liver function tests, respectively (Figure 2 and Figure 3).

### 3.4. Patterns in EQA Relative Deviation Distribution and the Relationship to NATA Class Prediction

While GGT was the leading predictor of NATA (ISO 15189) performance for the laboratory network examined here, it is likely that results from other laboratory EQA-ISO 15189 processes will identify different markers. To assist in the identification of EQA predictors in general, studies on relative deviation distribution frequencies were conducted to detect patterns associated with machine learning prediction of NATA results.

As well as GGT, another LFT marker, Total Protein (TP), was investigated as an example of a poor NATA outcome predictor.

**Figure 7:**
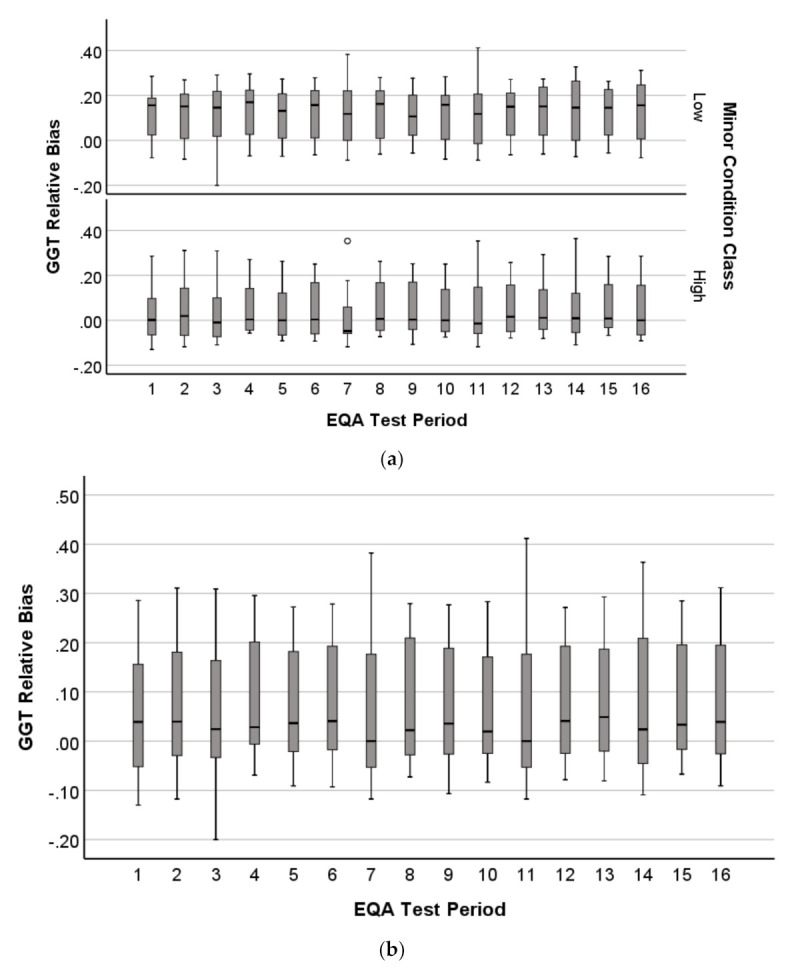

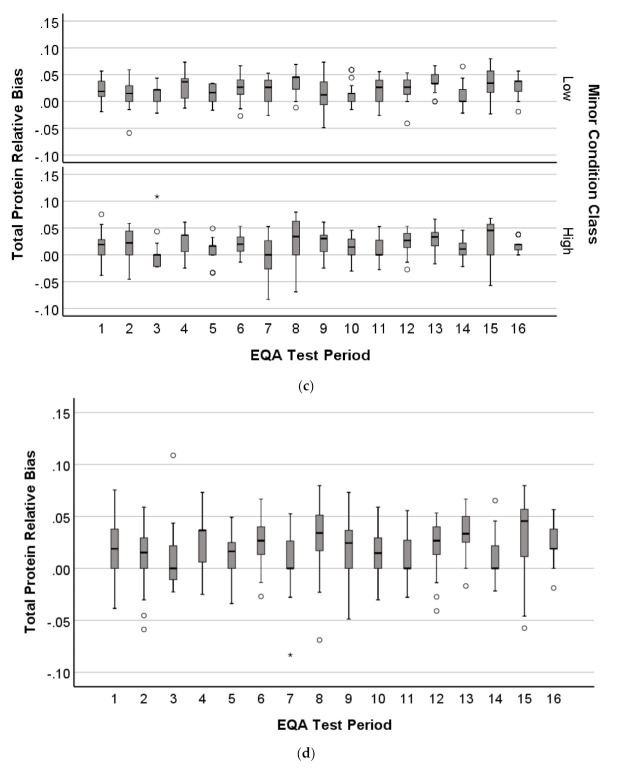
EQA relative deviation (bias) variation over a period of 16 EQA challenges for (**a**,**b**)-glutamyl transferase (GGT) and (**c**,**d**) Total Protein (TP), represented by boxplots (median ± interquartile ranges). Both GGT (**a**) and TP (**c**) were separated by NATA Minor Classes for variation comparison.

The boxplot medians for GGT showed distinct patterns depending on whether associated with high or low NATA Minor reporting (Figure 7a). Unexpectedly, the high NATA report class had medians consistently close to the EQA target (0.0 relative deviation), suggesting superior EQA performance, whereas low NATA report class consistently showed median trends close to 0.20 relative deviation. The combined GGT sample had steady EQA median relative deviations 0.0–0.10 (Figure 7b).

The TP boxplots did not show a dichotomy in relative deviation median variation over the 16 EQA tests dependent upon NATA high or low minor condition reporting, with both classes consistently varying 0.0 to 0.05 relative deviation. The combined sample showed the same narrow variation in medians (Figure 7c,d).

**Figure 8:**
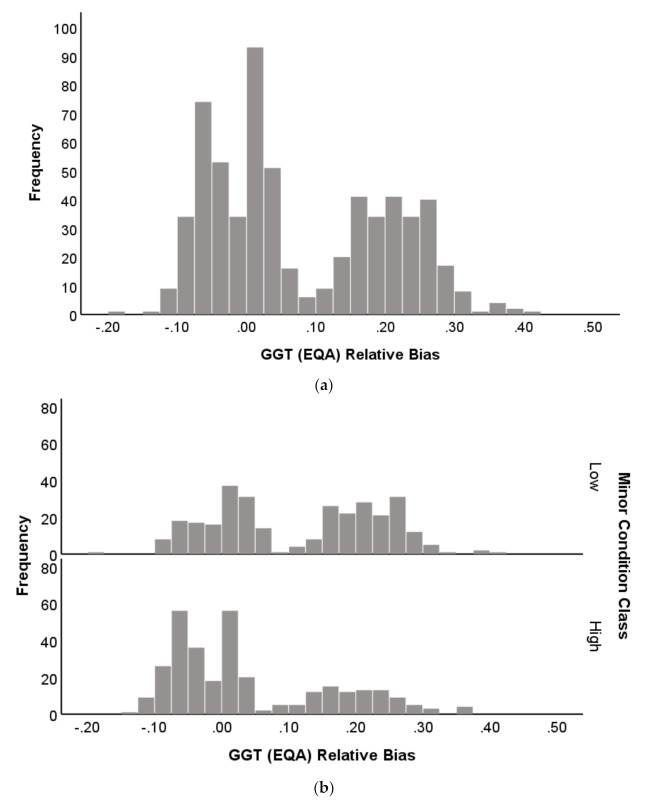

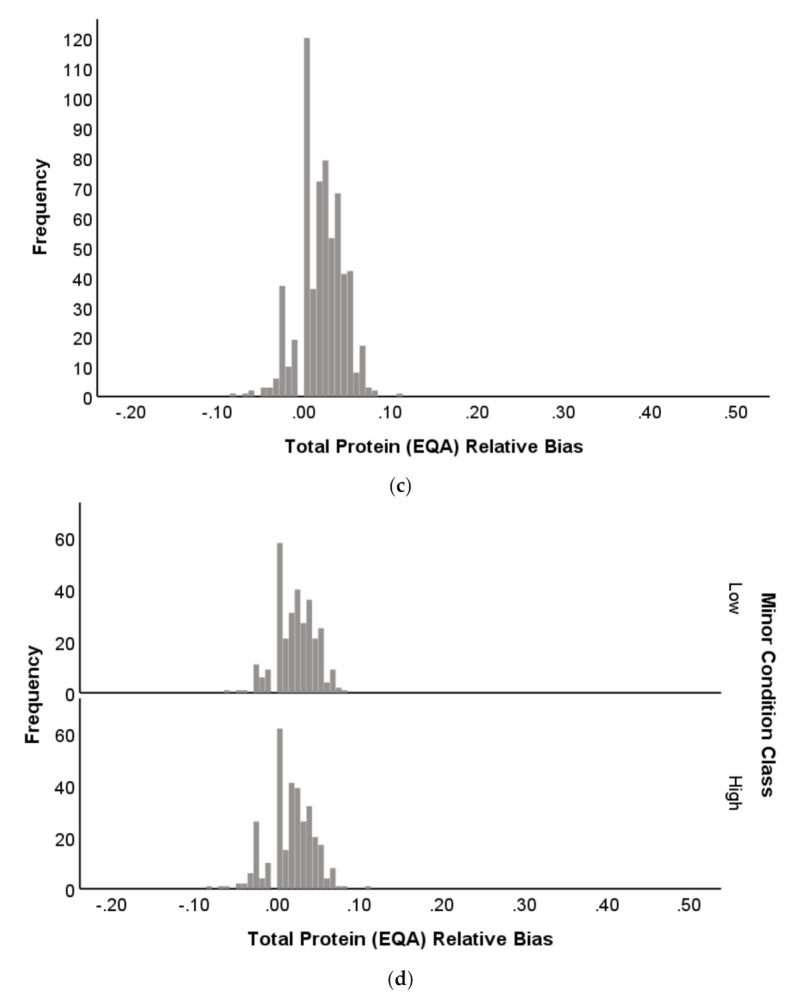
Frequency histograms for strong or poor EQA predictors of NATA Minor Condition Classes (High or Low)—(**Strong**—(**a**,**b**)) GGT and (**Poor**—(**c**,**d**)) Total Protein. The *x*-axis represents the distribution of cases across the range of analyte relative deviations calculated.

Histograms of EQA relative deviation for GGT and TP emphasized further the contribution of distribution to prediction performance (Figure 8a–d). The GGT histogram showed a bimodal distribution around an EQA relative deviation of approximately 0.10, and range from −0.20 to 0.40. The TP distribution was unimodal and had a narrower range (−0.10 to 0.10), indicating less variation.

Supporting these observations were the result of two-sample Kolmogorov–Smirnov tests, comparing the distributions of GGT and TP after separation by NATA (Minor) class (Figure 8b,d). The GGT distributions were significantly different (Kolmogorov–Smirnov Z = 3.898; 2-tails; *p* < 0.001), while significance was not achieved for TP (Kolmogorov–Smirnov Z = 1.010; 2-tails; *p* = 0.259). These results suggest that wider variation for EQA relative deviation in general, as well as when differentiated by NATA class, contributes mathematical properties to enhance NATA performance prediction by EQA analytes.

## 4. Discussion

Ensuring a consistent quality of clinical laboratory results is an ongoing endeavor, and while bolstered by the advent of ISO15189 standards [17,18,19,20,21,22], requires continuing vigilance due to issues such as laboratory and individual compliance [2,3], and modern pressures of a high-throughput system. In the Australian context, efforts via the RCPAQAP continue, in part, due to the recognition that traditional laboratory guidelines were conceived in the age prior to high-throughput platforms and computerization. Contemporary efforts include, for example, the evaluation of quality indicators (QIs) delivered through a Key Incident Monitoring and Management System (KIMMS), to understand laboratory quality management [23]. Additionally, the computerization of laboratory medicine presented new challenges associated with professional relationships between the laboratory and the clinic, and the dominant position of a “black-box” in the form of analytical platforms conducting analyses of patient samples [24].

In spite of these challenges to quality management posed by modern analytical platforms, the positive exploitation of mass data, AI and computational power were not as yet fully realized, as previously reported [6]. On surveying the pathology quality literature, only one reference was found that included the concept of computer-based systems to improve ISO15189 compliance, which was described in the context of training [25], but there is discussion on the application of AI in the context of healthcare broadly [26].

The application of machine learning to laboratory medicine has not been avoided altogether, with several studies successfully optimzsing and applying recursive partitioning and/or support vector machines (SVM) to pathology results for the assessment of HBV antiviral treatments, the prediction of hepatitis B virus (HBV) infection, and the assessment of liver function test efficiency [27,28,29,30,31]. The promise of digital histology results and linked data is also attracting attention, including in relation to ISO15189 compliance [32,33,34,35]. In the context of quality management for clinical laboratories, the potential of ML to produce new insights from the interrogation of the total quality system, represented for this study by integrated EQA-NATA results, was compelling, and supports the call by some for the “flexible scope …” of ISO 15189 accreditation [22].

The machine learning conducted for this study utilized two algorithms-recursive partitioning (decision trees and random forests), and support vector machines (SVM). These are not traditional statistical methods, but rely upon training and testing the model via the algorithm employed. For recursive partitioning this relies upon purity/impurity of outcome in the training data to determine how to split (“partition”) predictor variables into decision categories, underpinned by Entropy or Gini functions to assess purity [36,37]. Single decision tree models are taken further in the combination of tree predictors into ensembles (forests), and majority voting to predict the outcomes [38]. The SVM operates on a different foundation through the placement of support vectors and kernels into separate categories, which are initially mapped to a high-dimension input space, where after a linear decision (feature) space is presented [39]. These methods were conceived to solve classification problems, with regression modes also available for predictive modelling.

Given the complexity of the NATA and EQA data, illustrated by the lack of an obvious relationship (Table 1), the availability of extra classification power was valuable. Each method provides advantages that in combination allows an enhanced understanding of the problem of interest; clear decision boundaries from single trees, powerful classification and predictor identification via forests, and the capacity to define and generalize results with only two or three predictor variables via SVM.

The models presented herein required the calculation of relative deviation for each EQA analyte (to estimate specific target value accuracies), with the frequency of NATA Minor (M) reports used to establish nominal classes reflecting the extent of ISO15189 compliance by laboratories. The ensuing ML models were assessed in terms of the capacity to predict NATA class from EQA results obtained from the same assessment period. Reports by NATA assessors represent technical and/or management problems at the laboratories assessed, and may reflect in the EQA results as difficulties in obtaining accuracy. Hence, the NATA-EQA modelling could link systemic operational problems detected by NATA, with EQA performance at the analyte-detection level. Linkage of these data, therefore, may assist laboratories identify system-level issues in advance of NATA site audits, resulting in fewer Condition reports on subsequent NATA assessment.

GGT was the top-ranked EQA predictor of NATA class in all models examined. Furthermore, the inclusion of GGT in a creatinine-electrolyte model enhanced predictive performance by 4.5–6.0% (Table 3). In a combined LFT-Creatinine-Electrolyte model, GGT was followed by AST and ALT, and then creatinine and bicarbonate in the ranking of EQA markers as NATA predictors (Figure 3). These performances as predictors are not related to their biochemical functions physiologically, but to their variability between laboratories when attempting to achieve a result close to the EQA target value. This suggests that variability in the markers is due to issues of calibration, technical operation, assay reagents or overall laboratory management, which will reflect in the performance consistency between EQA cycles, and hence influence its potential for NATA outcome predictions. It appears that GGT is most sensitive to these fluctuations, with a definitive explanation of this sensitivity only speculative at this time. Supported by two-sample K–S tests, the variation of EQA relative deviation, as summarized here in boxplots and histograms, may explain the potential of specific EQA markers to act as superior predictors of NATA performance. Broader variation and the bimodal distribution of GGT relative deviation contrasted clearly with the poor NATA predictor, total protein (TP).

The NATA and EQA assessment and reporting systems do not match in method or timeframe, and as such, introduce a limitation. The NATA process involves an inspection of the laboratory every two years by assessors, who subsequently write reports and make recommendations on technical and management practices, and how non-compliance to standards may impact performance quality [7]. If there are concerns about management or technical standards, Conditions (serious or minor), can be imposed. This is a thorough and labor-intensive process that cannot be implemented on a regular basis. This contrasts with the EQA, which in Australia sends samples to laboratories on approximately a weekly basis, for the range of routine and special test markers. These assessments gauge quality simply through assay performance as measured by proximity to an assay-specific target value. For these reasons, it is impossible to directly match the results from one quality system with the other. However, the implications of ISO 15189 compliance performance, as assessed by NATA, evaluate the deep management and technical systems that support day-to-day performance, as captured by the RCPAQAP EQA process, and as such represent slow moving management change that may not realize change in the laboratories for months.

Taken together, the results presented herein provide direction on integrating NATA assessment results with the outcomes from the EQA process, and reveal details on what aspects of the models were most predictive of laboratory performance. Therefore, in designing an integrated quality assessment model (and eventually system) for the timely detection of problems encountered by clinical laboratories, it is recommended that EQA results are converted to a relative deviation value, plotted in a histogram and thereafter examined for a bimodal distribution. The detection of specific EQA markers to track individual laboratory networks in relation to quality assurance via ISO15189 will be further assisted by the application of random forest algorithms, assisted by single decision trees and SVMs to identify precise decision boundaries, and overall prediction patterns, respectively.

## Figures and Tables

**Table 1:** EQA quartile (%) rankings of laboratories according to: (**a**) serum Creatinine, (**b**) GGT and (**c**) serum Potassium, and matched with the Mean ($\bar{x}$) NATA conditions for each quartile (descending from the highest EQA performance to the lowest).

EQA Marker	Percent (%) Quartile	NATA Conditions Quartile Means $(\bar{x}$) (± SEM)		
		Minor (M)	Major (C)	Minor + Major
Serum Creatinine	1	3.6 ± 0.9	2.0 ± 1.0	5.6 ± 1.7
	2	6.4 ± 0.9	3.8 ± 0.6	10.1 ± 1.4
	3	7.7 ± 1.4	4.2 ± 1.3	11.9 ± 2.6
	4	4.8 ± 1.0	1.0 ± 0.6	5.8 ± 1.3
GGT	1	6.6 ± 1.0	5.1 ± 0.9	11.6 ± 1.9
	2	6.3 ± 1.4	3.0 ± 1.0	9.3 ± 2.2
	3	4.0 ± 0.9	1.7 ± 0.5	5.7 ± 1.2
	4	7.1 ± 1.4	2.5 ± 1.3	9.6 ± 2.5
Serum Potassium	1	6.3 ± 1.0	3.5 ± 0.9	9.8 ± 1.8
	2	3.8 ± 0.9	1.9 ± 1.0	5.7 ± 1.8
	3	7.3 ± 1.7	4.3 ± 0.9	11.5 ± 2.3
	4	6.4 ± 1.2	3.0 ± 1.0	9.4 ± 2.2

Percent Quartile Ranges: 1 (1–25%), 2 (26–50%), 3 (51–75%), 4 (76–100%). Quartile range 1 = Best EQA Performance; Quartile range 4 = Worst EQA Performance. Rankings determined after coefficient of variation percent (CV%) calculation, with the smallest CV% representing the best performance across the 16 time point EQA challenge, for the EQA marker concerned. M-Minor condition reported by NATA. C-major Condition reported by NATA.

**Table 2:** Predictive statistical and model tuning parameters of the random forest analyses presented in Figure 2, which interrogated serum creatinine-electrolyte + GGT (relative deviation) data for the best predictors of NATA class (High or Low Minor Condition reports).

Features and Results from RF Models *	Random Forest (RF)			
	Optimal mtry	Sensitivity	Specificity	Final Model Accuracy
Model Tuning	4	0.74	0.65	0.71 (95% CI: 0.64, 0.78)
Final Model Statistics	McNemar’s	Sensitivity	Specificity	
	0.27	0.77	0.65	
	Kappa	PPV	NPV	
	0.42	0.71	0.72	

* Model interrogated: NATA Minor (High/Low) Class → (EQA relative deviation results): Bicarbonate + Calcium + Chloride + Creatinine + Magnesium + Phosphate + Potassium + Sodium + GGT. The median number of Minor (condition) reports across all laboratories was calculated to allow the designation of high (>median) and low (<median) NATA Classes for modelling with EQA results. The McNemar’s result is a *p*-value, with *p* > 0.05 indicating no significant difference between the marginal totals from the 2 × 2 contingency table used for class prediction. Kappa statistic results are a proportion between 0.0–1.0 indicating degree of agreement between class predictions, with values 0.0–0.20 as no to slight agreement, 0.21–0.40 as fair, 0.41– 0.60 as moderate, 0.61–0.80 as substantial, and 0.81–1.00 perfect agreement. ROC (Receiver Operating Curve); PPV (Positive Predictive Value); NPV (Negative Predictive Value).

**Table 3:** The impact of GGT on the Electrolyte-Creatinine EQA model of NATA class prediction by random forest modelling.

NATA Class- Minor Reports	GGT Included in UEC Model		GGT Excluded from UEC Model	
	Prediction Accuracy (%)	Overall Accuracy (%)	Prediction Accuracy (%)	Overall Accuracy (%)
High (>Median)	241/320 (75.3%)	71.3%	230/320 (71.9%)	66.4%
Low (<Median)	198/296 (66.9%)		179/296 (60.5%)	

Model interrogated: NATA Minor (High/Low) Class → (EQA relative deviation results): serum Bicarbonate + Calcium + Chloride + Creatinine + Magnesium + Phosphate + Potassium + Sodium + GGT. The median number of Minor (condition) reports across all laboratories was calculated to allow the designation of high (>median) and low (<median) NATA Classes for modelling with EQA results.

**Table 4:** Predictive statistical and model tuning parameters of the random forest analyses presented in Figure 3 that interrogated LFT (relative deviation) data for the best predictors of NATA class (High or Low Minor Condition reports).

Features and Results from RF Models *	Random Forest (RF)			
	Optimal mtry	Sensitivity	Specificity	Final Model Accuracy
Model Tuning	4	0.75	0.71	0.72 (95% CI: 0.65, 0.78)
Final Model Statistics	McNemar’s	Sensitivity	Specificity	
	0.27	0.77	0.66	
	Kappa	PPV	NPV	
	0.43	0.71	0.73	

* Model interrogated: NATA Minor (High/Low) Class → (EQA relative deviation results): ALT + AST + TBil + GGT + LD + TP. The median number of Minor (condition) reports across all laboratories was calculated to allow the designation of high (>median) and low (<median) NATA Classes for modelling with EQA results. The McNemar’s result is a p-value, with *p* > 0.05 indicating no significant difference between the marginal totals from the 2 × 2 contingency table used for class prediction. Kappa statistic results are a proportion between 0.0–1.0 indicating degree of agreement between class predictions, with values 0.0–0.20 as no to slight agreement, 0.21–0.40 as fair, 0.41–0.60 as moderate, 0.61–0.80 as substantial, and 0.81–1.00 perfect agreement. ROC (Receiver Operating Curve); PPV (Positive Predictive Value); NPV (Negative Predictive Value).

**Table 5:** Statistical and final predictive model tuning parameters for the random forest analyses presented in Figure 3 that interrogated the combined serum creatinine-electrolyte + LFT (EQA relative deviation) data, to determine the best predictors of NATA class (High or Low Minor Condition reports).

Features and Results from RF Models	Random Forest (RF)			
	Optimal mtry	Sensitivity	Specificity	Final Model Accuracy
Model Tuning	2	0.73	0.66	0.71 (95% CI: 0.64, 0.78)
Final Model Statistics	McNemar’s	Sensitivity	Specificity	
	0.055	0.80	0.61	
	Kappa	PPV	NPV	
	0.42	0.69	0.74	

Model interrogated: NATA Minor (High/Low) Class → (EQA Relative Deviation results): serum Bicarbonate + Creatinine + GGT + ALT + AST. The median number of Minor (condition) reports across all laboratories was calculated to allow the designation of high (> median) and low (< median) NATA Classes for modelling with EQA results. The McNemar’s result is a p-value, with *p* > 0.05 indicating no significant difference between the marginal totals from the 2 × 2 contingency table used for class prediction. Kappa statistic results are a proportion between 0.0–1.0 indicating degree of agreement between class predictions, with values 0.0–0.20 as no to slight agreement, 0.21–0.40 as fair, 0.41–0.60 as moderate, 0.61–0.80 as substantial, and 0.81–1.00 perfect agreement. ROC (Receiver Operating Curve); PPV (Positive Predictive Value); NPV (Negative Predictive Value).

## Data Availability

Data available on request due to privacy restrictions.

## Supplementary Materials

The RCPAQAP protocol for calculating the coefficient of variation is available online at https://www.mdpi.com/article/10.3390/diagnostics11040692/s1.
