# Supplementary material for: Gamma-Glutamyl Transferase (GGT) Is the Leading External Quality Assurance Predictor of ISO15189 Compliance for Pathology Laboratories
Source: Diagnostics (Basel). 2021 Apr 13;11(4):692. doi: 10.3390/diagnostics11040692 (PMC8069573; doi:10.3390/diagnostics11040692)
Supplement: Supplementary file 1 [file diagnostics-11-00692-s001.pdf]

Supplementary Materials - *(Lidbury, Koerbin, Richardson, Badrick)*
Gamma-Glutamyl Transferase (GGT) is the Leading External Quality Assurance Predictor of ISO15189 Compliance for Pathology Laboratories

RCPAQAP Calculation of the Coefficient of Variation (%) and Other Quality Assurance Statistics

Precision and Accuracy examines the consistency of a test method across many samples. To do this, we can produce a linearity graph, which plots a participant's result for each sample on the y-axis, against the expected (median) result for that sample on the x-axis. It's then easy to see the distribution of results around the 'line of agreement', which is the 45º diagonal where the measured result equals the median (target) result.

Rules to perform linear regression analysis:

1. If the target source is considered when performing linear regression;

a. If the target source is MEDIAN of ALL results, linear regression uses the calculated median of ALL results to perform the comparison
b. If the target source is MEDIAN of the Category value, linear regression uses the calculated median of category results. If the range of results selected to perform linear regression contains less than 6 results in the data set for any sample, then the calculated median of ALL results is used to perform linear regression
c. If the target source is Specific target, linear regression uses the known target to perform linear regression

2. The selected sample range for the participant must contain the same method category values in all categories. Alternately, the participant may choose to view other method categories (via analytics functionality);

3. The minimum dataset or samples to perform linear regression is "6 samples". If less than 6 samples, linear regression is unable to be performed against the method category value, linear regression is based against ALL result median values.

Once the regression line is calculated, the precision and the accuracy can be described by high and low values, Standard Deviation (SD), Coefficient of Variation (CV%), and Average Bias. These can also be compared to other participants.

- Hypotheticals are determined from the value of the regression line at the lowest and highest targets (medians) in **Low and High Values** the group *(using the median of ALL results or the median low and high values of the category value groups).* These are a projection of the method's average performance, and will likely be different from the participant's actual highest and lowest results.
- The **Standard Deviation (SD)** reflects the standard error of the points on the regression line. It is proportional to the average distance of each point to the line of best fit, and represents how closely all of the results fit a line. Plots with points widely spread out will have a high SD, whereas points all falling very close to a straight line will have a low standard deviation.
- The **Coefficient of Variation** is another way to describe the spread of results. It's calculated as the SD divided by the average/mean of the participant's range of concentrations, expressed as a percentage mid-point average. As explained above, the CV accounts for concentration and so may be a better assessment of imprecision.
- The **Average Bias** describes the average distance between the line of expectation (45º line) and the regression line, specifically the distances at the low, mid and high target values.
- Each of these statistics can be plotted on a bar relative to the 20th, 50th and 90th percentiles for all participants. For example, if the participant's average bias is 4.2 IU/L and the 20th percentile is 4.4 IU/L, the participant is more accurate (i.e. the line of best fit is closer to the line of expectation) than 80% of participants.
